# Infectome Landscape of Rodents and Shrews in Guangdong Province Reveals Diverse Pathogens with Zoonotic Potential in Wildlife

**DOI:** 10.3390/v18050584

**Published:** 2026-05-21

**Authors:** Yukun Lin, Fenxiang Li, Peiyu Liang, Yangzi Zhou, Lihua Zhang, Wudi Zhou, Yufeng Liang, Ruolan Yu, Wei Yang, Zhijian Zhou, Zeliang Wei, Jian He, Jingzhe Jiang, Huacheng Yan

**Affiliations:** 1 School of Public Health, Guangzhou Medical University, Guangzhou 511436, China; linyukun6@163.com (Y.L.); zhanglh0112@163.com (L.Z.); 2 Southern China Center for Disease Control and Prevention, Guangzhou 510507, China; lifenxiang_nb@163.com (F.L.); 19258487114@163.com (W.Z.); li.angyufeng@163.com (Y.L.); 17306664699@163.com (R.Y.); yangwei534@163.com (W.Y.); summygg@163.com (Z.Z.); weizeliang1995@163.com (Z.W.); 3 Jiangsu Province Engineering Research Center for Marine Bio-Resources Sustainable Utilization and College of Oceanography, Hohai University, Nanjing 210098, China; liangpeiyulpy@gmail.com; 4 Institute of Pathogen Biology, Chinese Academy of Medical Sciences & Peking Union Medical College, Beijing 102629, China; zhouyangzi@ipbcams.ac.cn

**Keywords:** rodents, shrews, infectome landscape, phylogenetic analysis, zoonotic potential

## Abstract

Rodents and shrews are important reservoir hosts due to their close association with human activities and their role in carrying various zoonotic pathogens. Recently, meta-transcriptomic sequencing has become a powerful tool for surveilling and screening novel pathogens from wild animals. However, many of these studies focused only on the diversity and genetic evolution of viruses from wildlife, while ignoring non-viral pathogens such as bacterial and eukaryotic microorganisms. Here, we performed a comprehensive infectome analysis of 227 tissue samples collected from 42 rodents and 16 shrews across six cities of Guangdong Province, China. We identified 34 viral families, including 23 mammalian viruses. Phylogenetic analysis revealed a henipavirus from the kidneys of shrews closely related to the Langya virus with potential infection risks to humans. Additionally, two potential pathogenic bacteria and 12 eukaryotic pathogens from six genera were found, showing clearer organ tropism than viruses. Interestingly, a moderate positive abundance correlation between *Usmuvirus newyorkense* and *Trichinella* suggested a potential virus–parasite association. We used machine learning models to evaluate the zoonotic potential of the obtained viruses, which indicated that 15 of 23 viral species were high risk for human infection. These findings provide important insight into the substantial zoonotic threat posed by pathogens circulating in wild small mammals in southern China and highlight the necessity for persistent wildlife pathogen surveillance.

## 1. Introduction

Rodents and shrews are critical reservoirs for a wide range of zoonotic pathogens that pose significant risks to human health [1,2]. Rapid urbanization and increasing human encroachment into wildlife habitats, together with the ecological adaptability and high fecundity of rodents and shrews, have elevated the risk of zoonotic pathogen spillover [2,3]. Previous research has found that the major rodent-borne zoonoses include salmonellosis, leptospirosis, Lassa fever, and hantavirus diseases [4]. The recent discovery of Langya henipavirus in shrews has further highlighted the importance of eulipotyphlans as carriers of high-risk pathogens [5]. Growing evidence indicates that viruses previously thought to be restricted to rodents or bats, such as coronaviruses, severe fever with thrombocytopenia syndrome virus (SFTSV), and hantaviruses, are also present in shrews, suggesting broader host ranges than previously recognized [6,7,8,9]. Asymptomatic carriage in these hosts facilitates long-term pathogen maintenance and environmental transmission and may subsequently lead to disease outbreaks through contaminated water, urine, or wildlife consumption [10]. Moreover, many zoonotic parasites rely on small mammals as intermediate hosts, positioning them as critical reservoirs that facilitate the amplification of pathogens and exchange among humans, livestock, arthropods, and wildlife [11].

In recent years, meta-transcriptomics has greatly advanced the characterization of viral diversity, with a growing number of virome studies reshaping our understanding of viruses associated with wild animals [12,13,14,15]. Infectome analysis based on meta-transcriptomic sequencing has emerged as an efficient and increasingly cost-effective approach for investigating the pathogen composition of wildlife species, enabling the simultaneous detection of RNA viruses, DNA viruses, bacteria, and eukaryotic microorganisms [3,16,17].

Owing to its frequent international exchanges and favorable climatic conditions, Guangdong Province has become a hotspot for emerging infectious diseases (EIDs), including SARS [18]. The region has also reported a relatively high incidence of hemorrhagic fever with renal syndrome (HFRS), with 93–328 cases reported annually between 2015 and 2021 [19]. Although previous wildlife-associated virome studies in Guangdong have provided important insights into viral diversity, they have largely overlooked non-viral pathogens such as bacteria and eukaryotic microorganisms. As a result, the broader pathogen composition, relative abundance, tissue distribution, and potential interactions among different pathogen groups in common reservoir hosts remain poorly understood.

Here, we conducted a meta-transcriptomic survey and comprehensive infectome analysis of 227 internal organ samples from rodents and shrews in Guangdong, including lung, liver, spleen, and kidney tissues. With these data, we systematically compared mammalian pathogens across major microbial groups, including RNA viruses, DNA viruses, bacteria, and eukaryotic pathogens, and characterized their distribution patterns. Furthermore, we assessed the tissue tropism of these pathogens, identified potential interactions among co-detected microorganisms, and used a machine learning model (ZoonoticRank) to predict the zoonotic potential of the identified viral sequences. These data provide valuable insights into the pathogen landscape of small mammals in Guangdong Province and offer a foundation for future surveillance and risk assessment of zoonotic diseases.

## 2. Materials and Methods

### 2.1. Sample Collection

Six representative cities across Guangdong Province, China—Huizhou, Shaoguan, Shanwei, Chaozhou, Zhanjiang and Guangzhou—were selected as densely populated residential areas. From 2017 to 2023, a total of 42 rodents and 16 shrews were intermittently captured using cage traps. All animals were euthanized by intracardiac injection of pentobarbital. A total of 227 tissue samples, including livers, spleens, lungs, and kidneys, were collected. Each tissue specimen was immediately placed into a self-prepared viral transport medium (VTM, pH 7.4 Hank’s balanced salt solution containing 1% bovine serum albumin, 100 U/mL penicillin, and 50 μg/mL streptomycin) and stored on dry ice until transfer to –80 °C freezers.

### 2.2. RNA Extraction, Library Preparation and Sequencing

Each tissue sample was homogenized on ice in 900 μL of lysis buffer using a tissue homogenizer, followed by total RNA extraction using the RNeasy Plus Universal Tissue Mini Kit (Qiagen, Hilden, Germany). The integrity of extracted RNA was assessed using the QIAxcel Advanced Instrument (Qiagen, Hilden, Germany), and only qualified RNA samples were used for library construction. Before library preparation, RNA samples were pooled according to sampling locations, broad host categories (rodents or shrews), and organ types. The pooled RNA was treated with the Ribo-off rRNA Depletion Kit (Vazyme, Nanjing, China) to remove host ribosomal RNA, and libraries were subsequently prepared using the VAHTS Universal V10 RNA-seq Library Prep Kit for Illumina (Vazyme, Nanjing, China), according to the manufacturer’s protocol. These libraries were then sequenced on the Illumina NovaSeq platform (Illumina, San Diego, CA, USA) using a paired-end 150 bp strategy, and the target output of each library was 10 Gb.

### 2.3. Processing of Sequencing Data

Adapter sequences, duplicate reads, and low-quality reads were first removed using fastp (v1.0.0, HaploX Biotechnology, Shenzhen, China). Low-complexity reads were subsequently discarded with PRINSEQ++ (v1.2.4, San Diego State University, San Diego, CA, USA). Residual ribosomal RNA (rRNA) reads were then filtered out by aligning the cleaned reads against the SILVA rRNA database using Bowtie2 (v2.5.4, University of Maryland, College Park, MD, USA). The non-rRNA reads were de novo assembled using MEGAHIT (v1.2.9, University of Hong Kong, Hong Kong, China), and contigs longer than 800 bp were retained. These contigs were further clustered using CD-HIT (v4.8.1, Burnham Institute for Medical Research, La Jolla, CA, USA) at a 97% global average nucleotide identity threshold.

### 2.4. Virus Discovery and Classification

Assembled contigs were simultaneously subjected to viral identification and classification using DIAMOND BLASTx (v2.1.11, University of Tübingen, Tübingen, Germany), geNomad (v1.11.0, Joint Genome Institute, Berkeley, CA, USA), and VirBot (v1.0, City University of Hong Kong, Hong Kong, China). DIAMOND BLASTx searches were performed against the NCBI non-redundant protein database (nr, February 2024 release) using an E-value cutoff of 1 × 10^−5^. Contigs whose best hits fell within the viral taxonomic domain were considered putative viral genomes. Both geNomad and VirBot, being specialized tools for virus classification, also contributed putative viral genomes from their positive results. Phylogenetic analysis was conducted based on conserved proteins: the RNA-dependent RNA polymerase (RdRp) for RNA viruses, the non-structural protein 1 (NS1) for *Parvoviridae*, ORF1 for *Anelloviridae*, and the L protein for *Paramyxoviridae*. Sequences were aligned with reference viral proteins retrieved from GenBank using MAFFT (v7.526, Kyoto University, Kyoto, Japan), and ambiguously aligned regions were removed using TrimAl (v1.4, Centre for Genomic Regulation, Barcelona, Spain). The best-fit substitution model was selected using the ModelFinder implemented in IQ-TREE (v2.3.6, University of Vienna, Vienna, Austria), and the maximum likelihood (ML) trees were constructed using IQ-TREE with 1000 bootstrap replicates. Similarity plot analysis was performed using SimPlot (v.3.5.1, Johns Hopkins University, Baltimore, MD, USA). To detect the infection levels of *Mammarenavirus wenzhouense* in the organs of the individual in pooled libraries, real-time quantitative reverse transcription PCR (RT-qPCR) was performed using the Hifair III One Step RT-qPCR SYBR Green Kit (Yeasen, Shanghai, China). Specific primers were designed based on the obtained genomic sequences.

### 2.5. Discovery and Classification of Bacterial and Eukaryotic Pathogens

For the identification of bacterial and eukaryotic pathogens, a conservative detection strategy was adopted. Marker genes, including RNA polymerase subunit beta (*RpoB*), heat shock protein 60 (*GroEL*), recombination protein A (*RecA*), and DNA gyrase subunit B (*GyrB*), were used for bacterial pathogen detection. Elongation factor 1-alpha (*EF1α*), cytochrome c oxidase subunit 1 (*cox1*), and cytochrome b (*Cytb*) served as marker genes for eukaryotic pathogens. Based on DIAMOND BLASTx results (E-value ≤ 1 × 10^−10^), contigs were considered reliable pathogen indicators only if they showed > 90% amino acid identity and > 150 aa alignment to marker gene sequences. These thresholds were used as stringent initial screening criteria to reduce false-positive detection from short or fragmented contigs and to ensure sufficient sequence information for reliable downstream taxonomic and phylogenetic analyses. All bacterial and eukaryotic marker sequences were further cross-validated against the complete genome database of NCBI RefSeq to ensure sequence reliability. Final pathogen identification was further confirmed by phylogenetic analysis following the procedures described above. Considering the phylogenetic tree topology, microbial strains with <97% amino acid identity to known sequences were classified as putative novel species.

### 2.6. Quantification of Pathogen Abundance

Pathogen abundance was quantified using Transcripts Per Million (TPM), a normalization metric that accounts for both gene length and sequencing depth [20]. Viral contigs and non-viral contigs were quantified separately using FastViromeExplorer (v2018.04, Virginia Tech, Blacksburg, VA, USA) and Salmon (v1.10.1, Carnegie Mellon University, Pittsburgh, PA, USA) [21]. To mitigate false positives from index hopping (a common issue in high-throughput sequencing where reads from one sample are incorrectly assigned to another), a relative abundance cutoff was applied for each sequencing lane: any contigs whose read count in a library was below 0.2% of its maximum read count within the same lane were treated as index hopping and removed.

### 2.7. Pathogen Community Structure and Statistical Analysis

Viral diversity and community composition were analyzed using R (v4.3.2, R Foundation for Statistical Computing, Vienna, Austria) and Microsoft Excel (v16.0, Microsoft Corporation, Redmond, WA, USA). Alpha diversity indices were calculated with the vegan package, and beta diversity patterns were visualized through principal coordinates analysis (PCoA) using ggplot2. Group differences were evaluated with permutational multivariate analysis of variance (PERMANOVA), with statistical significance defined as *p* < 0.05.

### 2.8. Inferring Zoonotic Potential

To evaluate the zoonotic potential of detected viruses, machine learning models ZoonoticRank (v1.0, MRC-University of Glasgow Centre for Virus Research, Glasgow, United Kingdom) were used. This tool integrates genomic features, including codon usage, nucleotide composition, amino acid patterns, and dinucleotide frequencies, to estimate cross-species transmission risk and prioritize pathogens for targeted surveillance. This approach optimizes surveillance strategies and enhances our ability to predict and mitigate potential outbreaks, thereby improving public health preparedness. Following previous studies, a threshold of 0.293 was applied to identify viruses with elevated zoonotic risk [22]. All figures were generated using R, GraphPad Prism (v10.1.2, GraphPad Software, San Diego, CA, USA), and Microsoft Excel.

## 3. Results

### 3.1. Overview of the Samples and Sequencing Data

**Figure 1:**
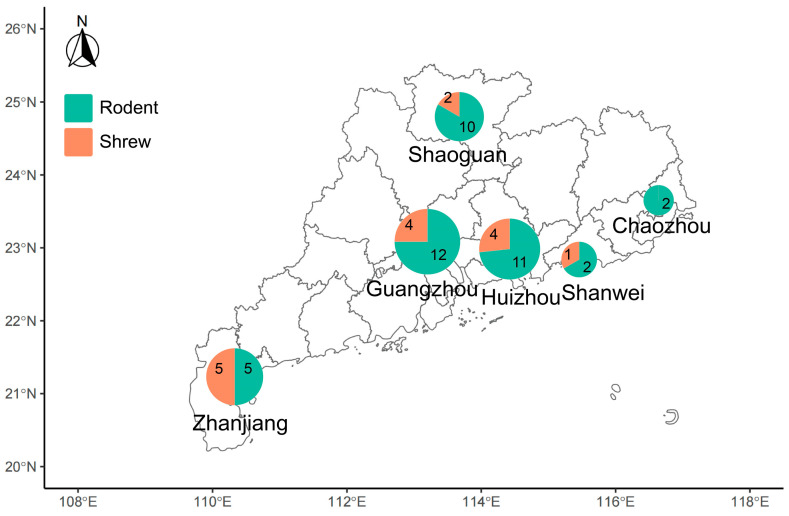
Geographic distribution of rodent and shrew samples collected from six cities in Guangdong Province. Pie charts indicate the numbers and proportions of rodents and shrews sampled at each location.

We performed a meta-transcriptomic investigation on 227 tissue samples (including lungs, livers, spleens and kidneys) collected from 42 rodents and 16 shrews in six cities of Guangdong Province, China. The six sampling sites represent five major geographical regions of Guangdong Province: east, south, west, north, and central (Figure 1). Samples were pooled based on host categories (rodents or shrews), tissue types, and geographical locations, and 51 libraries were constructed (Supplementary Table S1). A total of 3,866,608,428 raw reads were produced through paired-end sequencing. After filtering out low-quality and duplicate reads and removing residual rRNA reads, 5,276,996 de novo assembled contigs were obtained, with a median of 102,924 contigs per library (Supplementary Table S2). In the following sections, separate analyses of viral, bacterial, and eukaryotic pathogen diversity in rodents and shrews are presented.

### 3.2. The Diversity of the Virome

**Figure 2:**
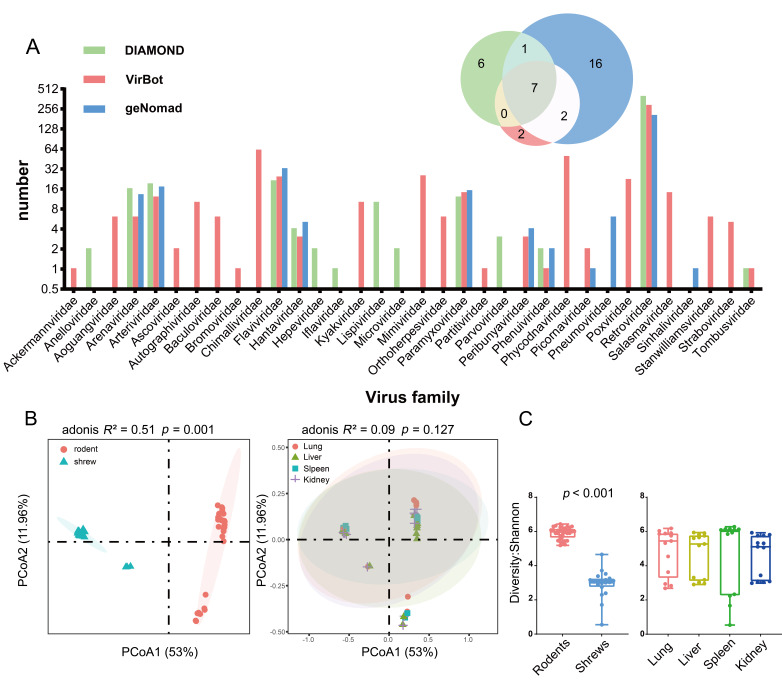
Diversity of viruses in rodents and shrews. (**A**) Counts of viral contigs classified at the family level by VirBot, geNomad, and DIAMOND, along with a Venn diagram showing the overlapping results from the three virus classification tools, representing the viral families they can detect. (**B**) PCoA of the virome composition across host categories and tissues. The left plot illustrates the separation of virome profiles between rodents and shrews. Statistical significance of the grouping was confirmed by PERMANOVA (*R*^2^ = 0.51, *p* = 0.001). The right plot shows the virome differences between different organs (liver, spleen, lung, and kidney). (**C**) Comparison of viral community alpha diversity across host categories and tissues. The comparisons were performed based on a Wilcoxon test (*p* < 0.001).

In order to comprehensively identify the viral contigs, we applied three widely recognized pathogen classification tools, DIAMOND, VirBot, and geNomad, combining sequence alignment and machine learning-based approaches to classify the assembled contigs, resulting a total of 2114 viral contigs. Only contigs assigned with family-level classifications were used for downstream analyses, ultimately retaining 1122 viral contigs belonging to 34 viral families (Figure 2A). Among the three tools, geNomad detected the largest number of contigs and the highest diversity at the family level, with a notable bias toward environmental viruses, protist viruses, and bacteriophages (*Baculoviridae*, *Mimiviridae,* and *Phycodnaviridae*). For major zoonotic viral families, including *Arteriviridae*, *Arenaviridae*, *Paramyxoviridae* and *Hantaviridae*, the three tools exhibited comparable detection performance (Figure 2A).

Diversity analysis revealed significant differences in viral composition between rodents and shrews, with rodents exhibiting higher viral species richness (Figure 2B,C). However, no statistically significant differences were found in viral composition among the four internal organs (Figure 2C). By merging results from read mapping (expressed as Transcripts Per Million, TPM) for contigs belonging to the same viral family, we compiled a final virome profile of small mammals in Guangdong (Supplementary Figure S1A). This profile revealed that rodents and shrews shared 23 viral families (Supplementary Figure S1B), many of which were detected across multiple regions, indicating their potential for long-term endemic circulation (Supplementary Figure S1A).

### 3.3. Identification and Characterization of Mammalian Viruses

**Figure 3:**
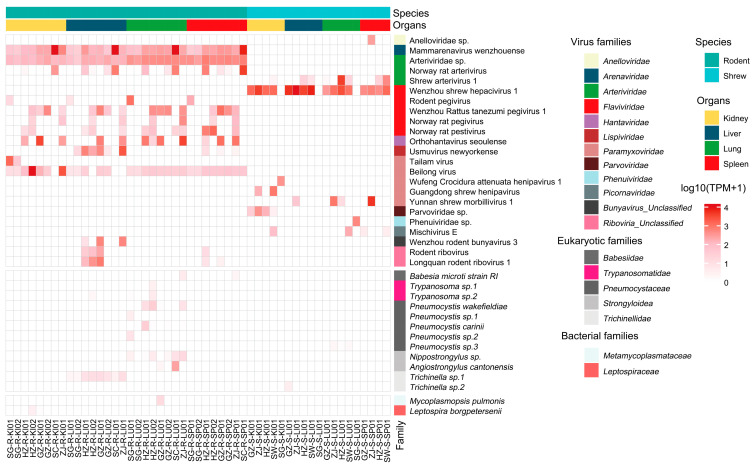
Diversity of mammalian pathogens in rodents and shrews. The heatmap is based on the log_10_(TPM+1) level of mapped reads of mammalian pathogens in each library. Host categories (rodents or shrews) and organs (kidney, liver, lung, and spleen) are annotated with colors at the top. The names of viral, bacterial, and eukaryotic families are provided on the right.

**Figure 4:**
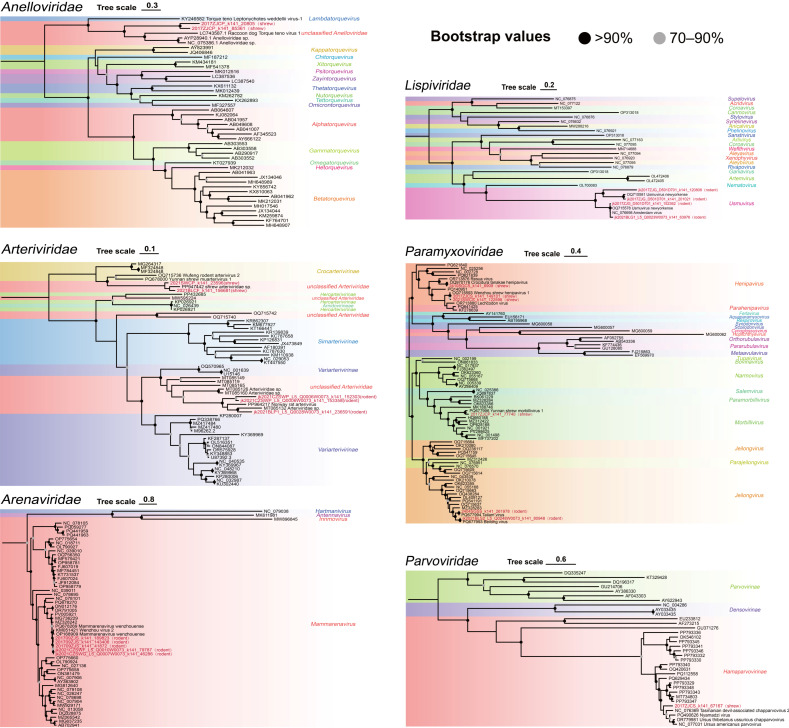

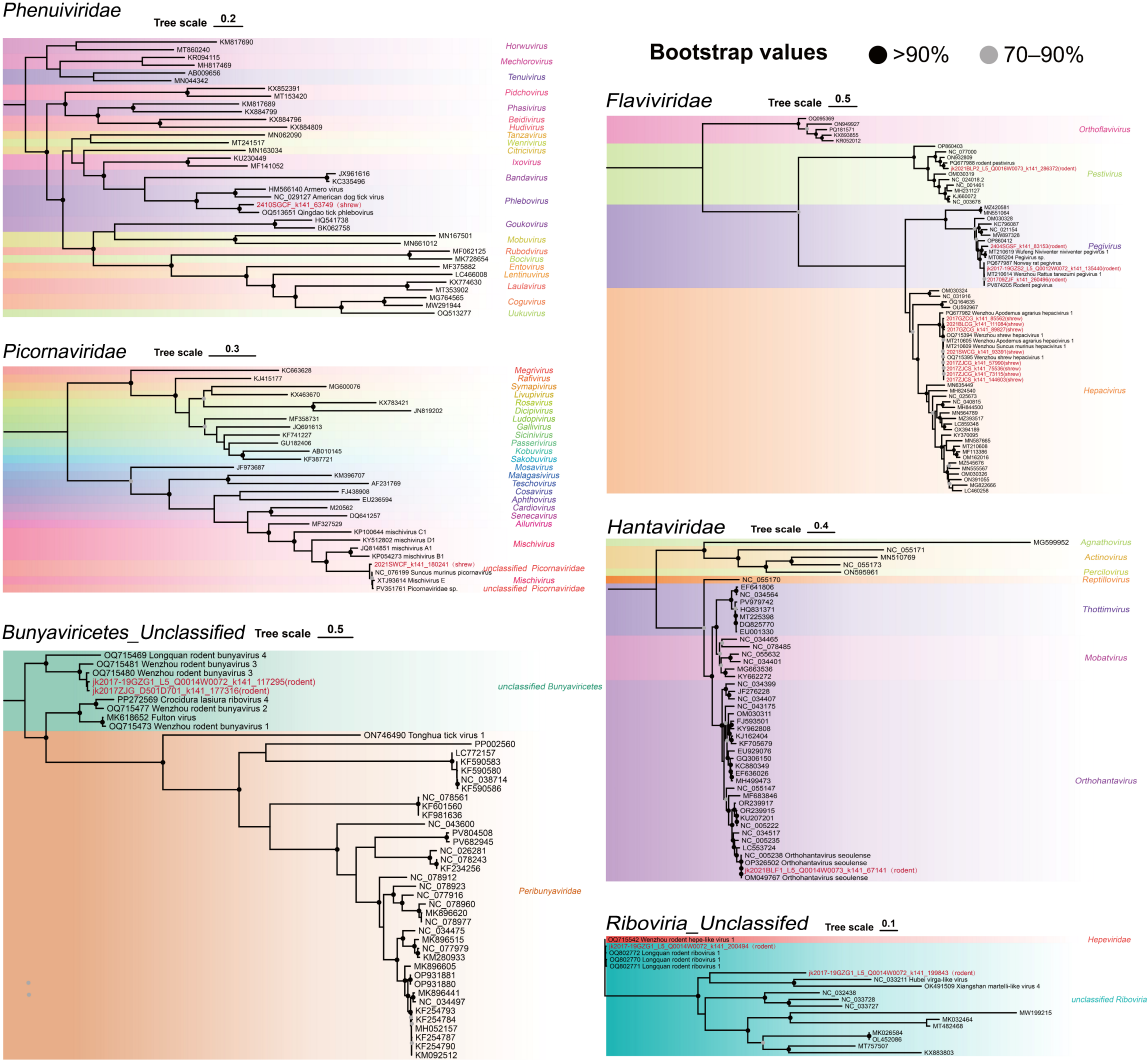
RNA and DNA viruses identified in this study. Maximum likelihood phylogenetic trees were estimated based on conserved proteins, namely the RdRP for RNA viruses, NS1 for the *Parvoviridae*, ORF1 for *Anelloviridae*, and the L protein for *Paramyxoviridae*. Red labels represent viruses detected in this study and are annotated with their host origins. Clades with viruses from the same genus/subfamily are shaded in matching colors.

Based on phylogenetic analyses of conserved proteins, 42 mammalian contigs were identified from the viral contigs and classified into 20 viral species spanning 10 viral families (Figure 3 and Figure 4). Given the current limitations in viral taxonomy, Longquan rodent ribovirus 1, Wenzhou rodent bunyavirus, and rodent ribovirus have not been assigned to specific viral families. However, due to the high similarity between the viral sequences obtained in previous studies and these viruses [12], they are included in the discussion. Ten viral contigs could be regarded as putative novel species (Supplementary Table S3). *Paramyxoviridae* and *Flaviviridae* exhibited the highest diversity, whereas *Mammarenavirus wenzhouense* and *Arteriviridae* sp. were detected in all rodent libraries and maintained high abundance across tissues and geographical regions (Figure 3).

**Figure 5:**
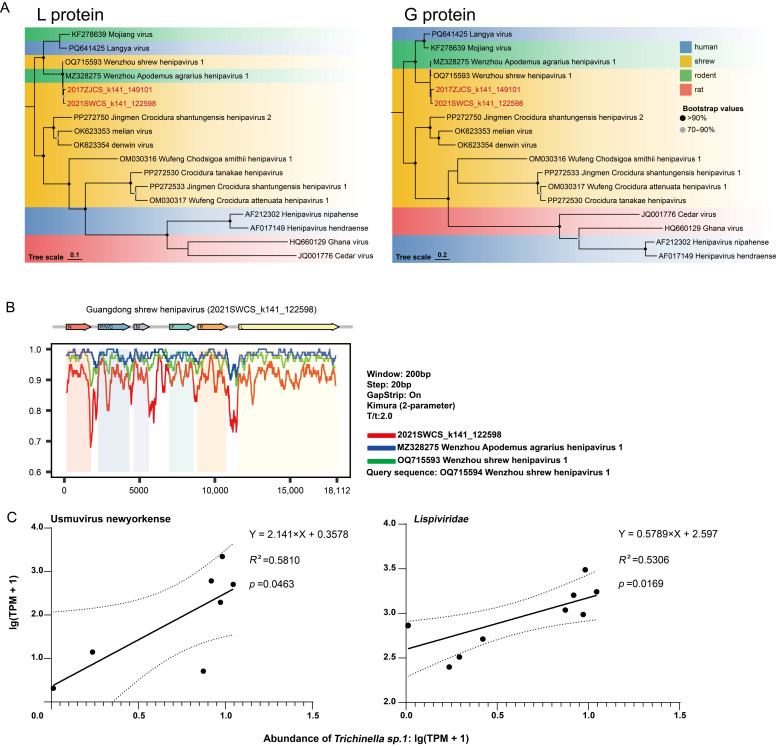
Genomic and phylogenetic characterization of a henipavirus species and the interactions among pathogens. (**A**) Maximum likelihood phylogenetic trees estimated using amino acid sequences of the L gene and the G gene within the genus *Henipavirus*. Color blocks indicate different species groups, and newly identified viruses are marked with red labels. (**B**) Genome organization of Guangdong shrew henipavirus (strain 2021SWCS_k141_122598) and SimPlot analysis of whole-genome nucleotide similarity with open reading frames (ORFs) depicted as colored arrows on the top. (**C**) Linear regression analysis between the abundance of the virus and the eukaryotic pathogen. Scatter plots with fitted regression lines show the relationship between the abundance (lg(TPM + 1)) of *Usmuvirus newyorkense* (*Lispiviridae*) and *Trichinella* sp.1.

Among the viruses identified, we focused on those with potential emergence risk based on their phylogenetic relationships to known human pathogens. A henipavirus sequence containing the complete coding region was identified in kidney libraries from Zhanjiang and Shanwei and was provisionally named Guangdong shrew henipavirus (strain 2021SWCS_k141_122598; accession no. C_AA280344.1). This sequence shared 84.9% amino acid identity in the L protein (large protein) and 67.6% in the G protein (attachment glycoprotein) with the Langya virus, which may underscore its potential risk as an emerging pathogen. Phylogenetic analyses further showed that Guangdong shrew henipavirus clustered with henipaviruses detected in diverse mammalian hosts, including human-associated viruses (Figure 5A). Interestingly, the rodent-borne Wenzhou Apodemus agrarius henipavirus 1 (MZ328275) shows closer whole-genome relatedness to Wenzhou shrew henipavirus 1 (OQ715593) than to Guangdong shrew henipavirus detected in shrews in our study (Figure 5B). This may suggest a recent cross-order transmission event within this clade.

The classical rodent-borne zoonotic viruses *Mammarenavirus wenzhouense* and *Orthohantavirus seoulense* were detected at high abundance and across multiple libraries, with *Mammarenavirus wenzhouense* identified in all rodent libraries (Figure 3). RT-qPCR screening of individual organs confirmed that the high abundance of arenaviruses predominantly originated from the 2021CZ01 individual, although other rodents also tested positive (Supplementary Table S4). The Ct values measured across different organs showed patterns consistent with TPM, supporting the reliability of meta-transcriptomic sequencing for viral quantification. Collectively, these results underscore the substantial burden of high-risk, potentially pathogenic viruses carried by rodents and shrews in Guangdong.

### 3.4. Discovery of Bacterial and Eukaryotic Pathogens

Through marker gene analysis, we identified 12 eukaryotic species from six genera (*Babesia*, *Trypanosoma*, *Pneumocystis*, *Nippostrongylus*, *Angiostrongylus*, and *Trichinella*) and two bacterial species (*Mycoplasmopsis* and *Leptospira*), all of which have pathogenic potential to humans (Supplementary Figures S2 and S3 and Table S5). Phylogenetic analyses identified previously uncharacterized members of the genera *Trichinella*, *Nippostrongylus* and *Trypanosoma*, which occupied distinct clades on the phylogenetic trees, indicating their unique evolutionary positions (Supplementary Figure S2).

We detected multiple eukaryotic pathogens in 23 (45%) of the libraries, with *Trichinella* and *Pneumocystis* among the most common (Figure 3). Additionally, zoonotic species such as *Babesia microti* and *Angiostrongylus cantonensis* were also identified. *Pneumocystis* exhibited the highest genetic diversity (Supplementary Figure S2), and its widespread occurrence further supports the growing recognition of coinfections involving multiple *Pneumocystis* species within a single host [23]. In comparison, bacterial pathogens were less common, with only *Leptospira borgpetersenii* (3.9%) and *Mycoplasmopsis pulmonis* (2.0%) detected (Figure 3).

Interestingly, *Usmuvirus newyorkense* and *Trichinella* sp.1 were frequently co-detected in the same library. We observed that the TPM values of *Lispiviridae* and its viral species, *Usmuvirus newyorkense*, each exhibited a linear correlation with those of *Trichinella* sp.1 (Figure 5C). Further transcriptomic profiling across five libraries demonstrated diverse gene expression patterns for *Trichinella*, indicating its active metabolic activity and true presence in the host (Supplementary Figure S4). This provides strong evidence supporting the reliability of the observed correlation between *Trichinella* and *Lispiviridae*.

### 3.5. Tissue-Specific Distribution of Pathogens

The distribution and abundance of mammalian pathogens were assessed across four tissues: lung, liver, spleen, and kidney. The lung exhibited the highest pathogen diversity (25 species), followed by the liver (19 species), kidney (19 species), and spleen (18 species) (Supplementary Figure S5A).

Overall, 29.7% (11/37) of the pathogens were shared among all four tissues, all of which were RNA viruses. These viruses primarily belonged to high-priority viral families, including *Arenaviridae*, *Hantaviridae*, *Flaviviridae*, and *Paramyxoviridae*, with most of them (9/11) detected in rodents (Figure 3). Other pathogens displayed distinct organotropisms. For example, Parvoviridae sp. was exclusively detected in the kidney and Anelloviridae sp. in the spleen (Figure 3). Compared with viruses, three eukaryotic pathogen genera and all bacterial species were exclusively detected in a single tissue type (Supplementary Figure S5B). Statistical analysis confirmed this pattern, showing that 85.7% (12/14) of non-viral pathogens were restricted to two or fewer organs, compared to only 34.8% (8/23) of viruses (Fisher’s exact test, *p* = 0.0055). These findings demonstrate that bacterial and eukaryotic pathogens exhibit significantly clearer organ tropism than viruses.

Three distribution patterns of pathogens were observed (Supplementary Figure S5B): (1) pathogens detected uniformly across all four organs with comparable abundance; (2) pathogens present in all organs but showing a clear abundance bias toward specific tissues; and (3) pathogens with an exclusive or strong preference for individual organs—a pattern particularly common among non-viral pathogens.

### 3.6. Identification of Viruses with High Zoonotic Potential

**Figure 6:**
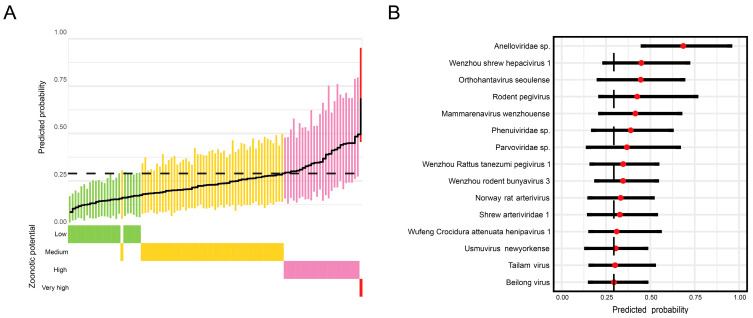
Zoonotic risk prediction. (**A**) Zoonotic risk prediction for mammalian viral contigs. Zoonotic risk prediction based on viral genomic features. Points represent mean calibrated scores, thick lines indicate the 95% confidence interval, and the black horizontal line denotes the cutoff value of 0.293. Colors represent assigned zoonotic potential categories: green, low risk; yellow, moderate risk; red, high risk; dark red, very high risk. (**B**) Taxonomic distribution of high-risk viruses. A total of 27 viral contigs were categorized as high or very high risk, belonging to 15 distinct viral species.

We employed the ZoonoticRank method to predict and rank the zoonotic potential of mammalian viral sequences. The ZoonoticRank analysis classified 26 viral sequences as high-risk and 1 sequence as very high-risk, originating from 15 different viral species (Figure 6A,B). The anelloviruses detected in this study received the highest predicted zoonotic risk scores. In addition, several rodent-borne viruses, including those represented by contigs closely clustered with *Orthohantavirus seoulense* and *Mammarenavirus wenzhouense* in the phylogenetic tree, were correctly identified as high-risk (Figure 4).

## 4. Discussion

In recent years, zoonotic pathogens originating from wildlife have become a major focus for public health. Two rodent viruses have been included in the “priority pathogens” list of the World Health Organization (WHO), and rodents are considered significant potential hosts for the hypothetical “Pathogen X” [24]. Traditionally, pathogen detection studies aimed at identifying threats to human health often collected samples such as oropharyngeal swabs, anal swabs, and feces to capture pathogen shedding routes and thereby assess spillover risk [25,26]. However, this approach may overlook pathogens circulating at low infection intensities within the hosts, particularly parasites [27]. The adoption of high-throughput sequencing has made it easier to comprehensively assess the pathogen carriage in mammals. In this study, we conducted a meta-transcriptomic analysis of major organs (lung, liver, spleen, and kidney) of rodents and shrews in Guangdong, China, and identified 34 viral families, including 23 mammalian viral species, 12 eukaryotic pathogens from six genera, and two potentially pathogenic bacterial species. We note that a similar study published in December 2025 characterized the pathogen spectrum of small mammals in Guangdong Province based on a larger sample size. While that study provides valuable baseline data for the region, our work differs in several important aspects, including the types of tissues analyzed, methodological strategies, analytical depth, and genome-level zoonotic risk evaluation. Therefore, our findings offer important complementary insights and further enrich the understanding of the infectome landscape of small mammals in Guangdong Province.

Among all the identified viral sequences, identification based on conserved proteins confirmed 23 viral pathogens related to mammals, although most were previously known species. Heatmaps of pathogens revealed specificity related to organ type and sampling location, but notable ubiquity was also observed (Figure 3 and Supplementary Figure S1A). For instance, *Mammarenavirus wenzhouense* was detected in all rodent libraries, indicating its potential for broad geographic circulation and strong capacity for zoonotic pathogen storage. Human serological surveys indicated that this virus was widespread in Southeast Asia, but viral RNA was rarely detected in related cases [28]. This discrepancy suggested that the virus had limited adaptability to humans compared with rodents, and human infection may have been underestimated. Consistent with previous virome studies, rodents displayed higher pathogen richness and diversity compared to shrews [29]. Phylogenetic analyses revealed distinct genetic divergence between the viruses in rodents and shrews (Figure 4). No evidence was found for simultaneous infection of the same virus species in both rodent and shrew groups, suggesting that host genetic differences were the primary driving factors for viral composition, despite their occupying similar ecological niches [3]. Therefore, the exchange of pathogens between rodents and shrews appeared limited, and no recent cross-species transmission events were observed in our study.

An important finding was the identification of the Guangdong shrew henipavirus, which clustered with henipaviruses previously detected in rodents, shrews, and humans, including the Langya virus, which causes severe diseases in humans [30]. This phylogenetic pattern indicates that the newly identified virus is related to a group of henipaviruses with broad host associations, but direct evidence of host-jumping for this specific virus is currently lacking (Figure 5A). Moreover, Guangdong shrew henipavirus is also phylogenetically close to the highly pathogenic Nipah and Hendra viruses, and the closely related Langya virus was detected in febrile patients in Eastern China in 2022 [31]. Remarkably, Wenzhou shrew henipavirus 1 (MZ328275) and Wenzhou Apodemus agrarius henipavirus 1 (OQ715593) exhibit high similarity, with their genomes sharing 97.1% nucleotide identity. Similarity plot analysis further confirmed that these strains detected in hosts from different orders were even more closely related to each other than to the Guangdong shrew henipavirus detected in shrews in this study (Figure 5B). These findings suggest that viruses within this clade may have the potential for cross-order transmission, and may reflect a transmission event occurring in Wenzhou, China, around 2016. Greater attention should be given to shrews as potential major reservoirs of henipaviruses.

For the identification of bacterial and eukaryotic pathogens, we focused exclusively on organisms of known or suspected relevance to human health. Several notable eukaryotic pathogens were detected: *Babesia microti*, the primary agent of human babesiosis in the United States, causes fever, hemolysis, and immunocompromise in humans [32]; *Trypanosoma cruzi*, the causative agent of Chagas disease, can cause chronic systemic infections leading to long-term cardiac damage; *Pneumocystis* exhibited high genetic diversity, clustering primarily with *P. wakefieldiae* and *P. carinii* lineages (Supplementary Figure S2). Although mixed *Pneumocystis* infections are known in some mammals, the ecological interactions and clinical significance remain uncertain [33]; *Angiostrongylus cantonensis* is a primary cause of human eosinophilic meningoencephalitis [34], with over 3000 global cases reported to date. Additionally, two distinct *Trichinella* lineages were found in rodents and shrews, each forming novel phylogenetic clades, suggesting the presence of previously undescribed species (Supplementary Figure S2). Although local parasitic disease prevention measures and health education are now sufficiently robust to maintain a low incidence of human trichinellosis [35], *Trichinella* still represents a persistent zoonotic pathogen as a major foodborne parasite that warrants our ongoing attention. Its high detection rate in rodents, along with the potential emergence of novel species, further underscores this evolving threat and highlights the need for sustained vigilance. Among bacterial pathogens, *Mycoplasma pulmonis*, a natural respiratory pathogen in rodents, has never been isolated from humans. However, experimental immunological evidence suggests its zoonotic potential [36], and prolonged contact with laboratory rats may lead to human infection [37]. *Leptospira borgpetersenii* is a recognized zoonotic pathogen, and rodents are the maintenance hosts for this species. Humans can be infected through various transmission routes, although the primary mode remains unclear [38]. Both *M. pulmonis* and *L. borgpetersenii* were detected in kidney libraries (Figure 3), indicating that infected individuals may have been actively shedding these pathogens. This underscores that rodent control programs and pathogen surveillance may mitigate disease transmission risks [39]. Collectively, these results demonstrate that bacterial and eukaryotic pathogens exhibit broader host ranges and rely on diverse maintenance and amplification hosts [40]. Their transmission can occur through multiple pathways, including direct contact, aerosols, contaminated food or water, and arthropod vectors [41]. The high diversity of parasites identified in this study highlights the need for long-term surveillance using infectome analysis, which can help address the gap in monitoring non-viral pathogens across wildlife hosts.

In addition, viruses within *Lispiviridae* are thought to primarily infect arthropods and nematodes [42], though some evidence suggests they can also infect mammals and birds [29]. The correlation analysis between the TPM values of *Trichinella* sp.1 and *Usmuvirus newyorkense* (*Lispiviridae*) revealed a moderate positive association. Given that previously reported Usmuviruses were detected in rodent samples and have been associated with parasitic nematodes [43], this preliminary observation may suggest a potential association between *Usmuvirus newyorkense* and *Trichinella* sp.1 in our study. However, this correlation alone is insufficient to confirm *Trichinella* as the true host of this virus or to demonstrate active viral replication. Further validation, such as detection of antigenomic RNA or in situ hybridization, is required to clarify the biological relationship between *Usmuvirus newyorkense* and parasitic nematodes. This finding highlights the potential value of infectome analysis for generating hypotheses about virus–parasite interactions (Figure 5C).

The distribution patterns of pathogens across different organs provide critical insights for guiding future surveillance efforts. The lungs exhibited the highest diversity, particularly for eukaryotic pathogens, aligning with findings from previous infectome studies [3]. The multi-organ infection with high abundance caused by high-priority viral families suggests that rodents, as reservoir hosts, maintain a substantial viral load across multiple organs, which could potentially spill over through respiratory, fecal–oral, and circulatory transmission routes (Figure 3). Compared to viruses, bacterial and eukaryotic pathogens exhibit more restricted tissue tropism (Supplementary Figure S5B). An initial characterization and summary of the organ infection distribution patterns of individual pathogens can help understand their potential transmission routes and inform the development of targeted surveillance strategies.

Although zoonotic potential was estimated solely based on genomic signatures, the ability of ZoonoticRank to accurately rank the zoonotic risk of known rodent-borne viruses suggests that its predictions may provide valuable insights for virus surveillance. The results revealed a higher number of high-risk viruses than previously anticipated. For instance, a novel anellovirus was ranked as the virus with the highest zoonotic risk by ZoonoticRank. However, phylogenetic analysis showed that it is distantly related to known human-infecting genera *Alphatorquevirus*, *Betatorquevirus*, and *Gammatorquevirus* (Figure 3). This divergence suggests that *Anelloviridae* may harbor a broader diversity of viruses with potential human pathogenicity than is currently recognized. Nevertheless, the lack of cell culture systems or animal models has limited our knowledge of this virus [44]. It must be acknowledged that current tools for predicting viral zoonotic potential are preliminary, and their accuracy requires additional confirmatory testing. We can achieve more targeted and effective interventions by identifying and prioritizing key host species that harbor viruses with the highest spillover potential through direct control measures and ecological approaches.

There are several limitations that need to be noted in our study. Firstly, the small sample size and uneven sampling across locations limited our confidence in assessing geographic differences in pathogen profiles. In addition, the unequal sampling effort between rodents and shrews may affect direct comparisons of pathogen diversity between the two host groups. Therefore, these diversity patterns should be interpreted as observations from this cross-sectional survey rather than broad ecological generalizations. Secondly, for many prevalent eukaryotic pathogens, the lack of sufficient reference genomes for comparison in existing databases necessitated the adoption of a highly conservative method for non-viral pathogen identification to minimize false positives. This may lead to inaccuracies in abundance quantification, and some previously reported parasites (*Toxoplasma gondii*) may have been missed [45]. Lastly, the zoonotic potential predicted by ZoonoticRank should be interpreted with caution. Although this tool is useful for preliminary risk prioritization, its predictions are mainly based on genomic signatures and cannot fully capture ecological exposure, host contact patterns, or molecular mechanisms required for cross-species transmission. Therefore, these scores do not provide definitive evidence of zoonotic infectivity. Further in vitro and in vivo studies are needed to directly evaluate the replication capacity, pathogenicity, transmissibility, and host range of the identified viruses.

## 5. Conclusions

This study provides a representative infectome landscape across four major organs (lung, liver, spleen, and kidney) for rodents and shrews in Guangdong. Our findings indicate that wild rodents and shrews in South China serve as important reservoirs of multiple high-risk pathogens with potential human pathogenicity and that a substantial proportion of the detected diversity likely represents previously uncharacterized agents that warrant further investigation. This study emphasizes the critical need for sustained wildlife pathogen surveillance in this region and lays the groundwork for more effective discovery and characterization of potential zoonotic pathogens.

## Data Availability

The raw sequencing data generated in this study have been archived in the China National Center for Bioinformation (CNCB) databases (www.cncb.ac.cn) under the GSA accession CRA042472. Furthermore, the viral genome sequences identified herein are accessible via GenBase (accession numbers C_AA280329.1 to C_AA280370.1). Any Supplementary Materials or additional datasets pertaining to this research are available from the corresponding authors upon reasonable request.

## Supplementary Materials

The following supporting information can be downloaded at https://www.mdpi.com/article/10.3390/v18050584/s1, Table S1: Information on sample group and RNA libraries in this study; Table S2: Summary statistics of assembled contigs from each meta-transcriptomic library; Table S3: Viruses identified in this study; Table S4: Detection of Mammarenavirus wenzhouense in various organs within individual rodents; Table S5: Summary of contigs with BLASTx hits to conserved bacterial and eukaryotic marker genes; Figure S1: Diversity of virome in rodents and shrews; Figures S2 and S3: Identification of bacterial and eukaryotic pathogens; Figure S4: 25 expressed genes in libraries with relatively high *EF1a* abundance (TPM > 5) of *Trichinella* sp.1; Figure S5: Tissue-specific distribution of pathogens.

## Abbreviations

The following abbreviations are used in this manuscript:

SFTSV	Severe fever with thrombocytopenia syndrome virus
EIDs	Emerging infectious diseases
SARS	Severe acute respiratory syndrome
HFRS	Hemorrhagic fever with renal syndrome
VTM	Viral transport medium
rRNA	Ribosomal RNA
BLAST	Basic Local Alignment Search Tool
NCBI	National Center for Biotechnology Information
RdRp	RNA-dependent RNA polymerase
NS1	Non-structural protein 1
RT-qPCR	Real-time quantitative reverse transcription polymerase chain reaction
*RpoB*	RNA polymerase subunit beta
*GroEL*	Heat shock protein 60
*RecA*	Recombination protein A
*GyrB*	DNA gyrase subunit B
*EF1α*	Elongation factor 1-alpha
*c**ox**1*	Cytochrome c oxidase subunit 1
*Cytb*	Cytochrome b
PCoA	Principal coordinates analysis
PERMANOVA	Permutational multivariate analysis of variance
L protein	Large protein
G protein	Attachment glycoprotein
WHO	World Health Organization
