# Supplementary material for: Infectome Landscape of Rodents and Shrews in Guangdong Province Reveals Diverse Pathogens with Zoonotic Potential in Wildlife
Source: Viruses. 2026 May 21;18(5):584. doi: 10.3390/v18050584 (PMC13211394; doi:10.3390/v18050584)
Supplement: Supplementary file 1 [file viruses-18-00584-s001.zip › Supplementary Figures S1-S5 .pdf]

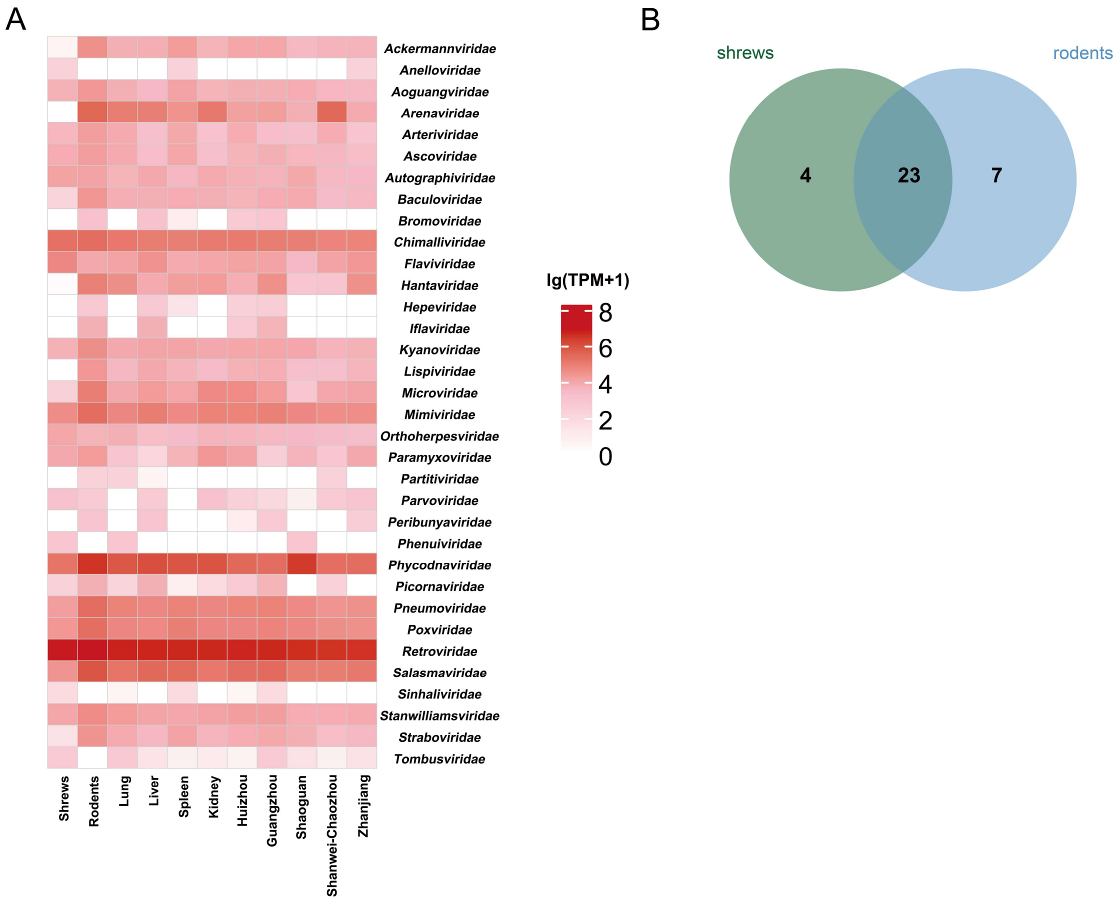

Supplementary Figure S1. Diversity of virome in rodents and shrews. (**A**) Heatmap showing the distribution of viral families across different host species, tissues, and sampling locations. (**B**) Venn diagram of viral families sharing across rodents and shrews.

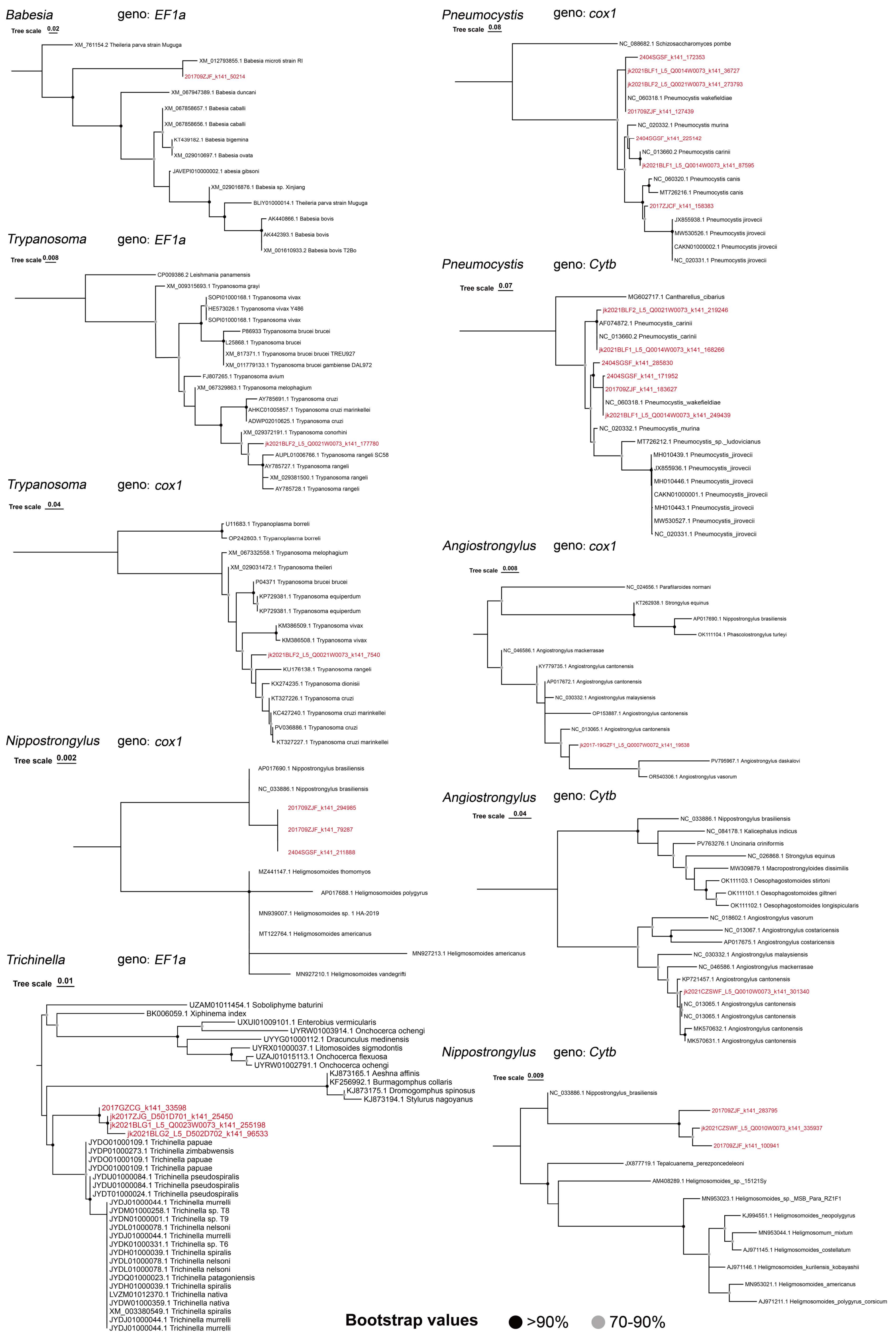

Babesia geno: EF1a
Tree scale 0.02
XM_761154.2 Theileria parva strain Muguga
XM_012793855.1 Babesia microti strain RI
201709ZJF_k141_50214
XM_067947389.1 Babesia duncani
XM_067858657.1 Babesia caballi
XM_067858656.1 Babesia caballi
KT439182.1 Babesia bigemina
XM_029010697.1 Babesia ovata
JAVEPI010000002.1 abesia gibsoni
XM_029016876.1 Babesia sp. Xinjiang
BLIY01000014.1 Theileria parva strain Muguga
AK440866.1 Babesia bovis
AK442393.1 Babesia bovis
XM_001610933.2 Babesia bovis T2Bo
Trypanosoma geno: EF1a
Tree scale 0.008
CP009386.2 Leishmania panamensis
XM_009315693.1 Trypanosoma grayi
SOPI01000168.1 Trypanosoma vivax
HE573026.1 Trypanosoma vivax Y486
SOPI01000168.1 Trypanosoma vivax
P86933 Trypanosoma brucei brucei
L25868.1 Trypanosoma brucei
XM_817371.1 Trypanosoma brucei brucei TREU927
XM_011779133.1 Trypanosoma brucei gambiense DAL972
FJ807265.1 Trypanosoma avium
XM_067329863.1 Trypanosoma melophagium
AY785691.1 Trypanosoma cruzi
AHKC01005857.1 Trypanosoma cruzi marinkellei
ADWP02010625.1 Trypanosoma cruzi
XM_029372191.1 Trypanosoma conorhini
jk2021BLF2_L5_Q0021W0073_k141_177780
AUPL01006766.1 Trypanosoma rangeli SC58
AY785727.1 Trypanosoma rangeli
XM_029381500.1 Trypanosoma rangeli
AY785728.1 Trypanosoma rangeli
Trypanosoma geno: cox1
Tree scale 0.04
U11683.1 Trypanoplasma borreli
OP242803.1 Trypanoplasma borreli
XM_067332558.1 Trypanosoma melophagium
XM_029031472.1 Trypanosoma theileri
P04371 Trypanosoma brucei brucei
KP729381.1 Trypanosoma equiperdum
KP729381.1 Trypanosoma equiperdum
KM386509.1 Trypanosoma vivax
KM386508.1 Trypanosoma vivax
jk2021BLF2_L5_Q0021W0073_k141_7540
KU176138.1 Trypanosoma rangeli
KX274235.1 Trypanosoma dionisii
KT327226.1 Trypanosoma cruzi
KC427240.1 Trypanosoma cruzi marinkellei
PV036886.1 Trypanosoma cruzi
KT327227.1 Trypanosoma cruzi marinkellei
Nippostrongylus geno: cox1
Tree scale 0.002
AP017690.1 Nippostrongylus brasiliensis
NC_033886.1 Nippostrongylus brasiliensis
201709ZJF_k141_294985
201709ZJF_k141_79287
2404SGSF_k141_211888
MZ441147.1 Heligmosomoides thomomyos
AP017688.1 Heligmosomoides polygyrus
MN939007.1 Heligmosomoides sp. 1 HA-2019
MT122764.1 Heligmosomoides americanus
MN927213.1 Heligmosomoides americanus
MN927210.1 Heligmosomoides vandegrifti
Trichinella geno: EF1a
Tree scale 0.01
UZAM01011454.1 Sobolipyhme baturini
BK006059.1 Xiphinema index
UXUI01009101.1 Enterobius vermicularis
UYRW01003914.1 Onchocerca ochengi
UYYG01000112.1 Dracunculus medinensis
UYRX01000037.1 Litomosoides sigmodontis
UZAJ01015113.1 Onchocerca flexuosa
UYRW01002791.1 Onchocerca ochengi
KJ873165.1 Aeshna affinis
KF256992.1 Burmagomphus collaris
KJ873175.1 Dromogomphus spinosus
KJ873194.1 Stylurus nagoyanus
2017GZCG_k141_33598
jk2017ZJG_D501D701_k141_25450
jk2021BLG1_L5_Q0023W0073_k141_255198
jk2021BLG2_L5_D502D702_k141_96533
JYDO01000109.1 Trichinella papuae
JYDP01000273.1 Trichinella zimbabwensis
JYDO01000109.1 Trichinella papuae
JYDO01000109.1 Trichinella papuae
JYDU01000084.1 Trichinella pseudospiralis
JYDU01000084.1 Trichinella pseudospiralis
JYDT01000024.1 Trichinella pseudospiralis
JYDJ01000044.1 Trichinella murrelli
JYDM01000258.1 Trichinella sp. T8
JYDN01000001.1 Trichinella sp. T9
JYDL01000078.1 Trichinella nelsoni
JYDJ01000044.1 Trichinella murrelli
JYDK01000331.1 Trichinella sp. T6
JYDH01000039.1 Trichinella spiralis
JYDL01000078.1 Trichinella nelsoni
JYDL01000078.1 Trichinella nelsoni
JYDQ01000023.1 Trichinella patagoniensis
JYDH01000039.1 Trichinella spiralis
LVZM01012370.1 Trichinella nativa
JYDW01000359.1 Trichinella nativa
XM_003380549.1 Trichinella spiralis
JYDJ01000044.1 Trichinella murrelli
JYDJ01000044.1 Trichinella murrelli
Pneumocystis geno: cox1
Tree scale 0.08
NC_088682.1 Schizosaccharomyces pombe
2404SGSF_k141_172353
jk2021BLF1_L5_Q0014W0073_k141_36727
jk2021BLF2_L5_Q0021W0073_k141_273793
NC_060318.1 Pneumocystis wakefieldiae
201709ZJF_k141_127439
NC_020332.1 Pneumocystis murina
2404SGSF_k141_225142
NC_013660.2 Pneumocystis carinii
jk2021BLF1_L5_Q0014W0073_k141_87595
NC_060320.1 Pneumocystis canis
MT726216.1 Pneumocystis canis
2017ZJCF_k141_158383
JX855938.1 Pneumocystis jirovecii
MW530526.1 Pneumocystis jirovecii
CAKN01000002.1 Pneumocystis jirovecii
NC_020331.1 Pneumocystis jirovecii
Pneumocystis geno: Cytb
Tree scale 0.07
MG602717.1 Cantharellus_cibarius
jk2021BLF2_L5_Q0021W0073_k141_219246
AF074872.1 Pneumocystis_carinii
NC_013660.2 Pneumocystis_carinii
jk2021BLF1_L5_Q0014W0073_k141_168266
2404SGSF_k141_285830
2404SGSF_k141_171952
201709ZJF_k141_183627
NC_060318.1 Pneumocystis_wakefieldiae
jk2021BLF1_L5_Q0014W0073_k141_249439
NC_020332.1 Pneumocystis_murina
MT726212.1 Pneumocystis_sp._ludovicianus
MH010439.1 Pneumocystis_jirovecii
JX855936.1 Pneumocystis_jirovecii
MH010446.1 Pneumocystis_jirovecii
CAKN01000001.1 Pneumocystis_jirovecii
MH010443.1 Pneumocystis_jirovecii
MW530527.1 Pneumocystis_jirovecii
NC_020331.1 Pneumocystis_jirovecii
Angiostrongylus geno: cox1
Tree scale 0.008
NC_024656.1 Parafilaroides normani
KT262938.1 Strongylus equinus
AP017690.1 Nippostrongylus brasiliensis
OK111104.1 Phascolostrongylus turleyi
NC_046586.1 Angiostrongylus mackerrasae
KY779735.1 Angiostrongylus cantonensis
AP017672.1 Angiostrongylus cantonensis
NC_030332.1 Angiostrongylus malaysiensis
OP153887.1 Angiostrongylus cantonensis
NC_013065.1 Angiostrongylus cantonensis
jk2017-19GZF1_L5_Q0007W0072_k141_19538
PV795967.1 Angiostrongylus daskalovi
OR540306.1 Angiostrongylus vasorum
Angiostrongylus geno: Cytb
Tree scale 0.04
NC_033886.1 Nippostrongylus brasiliensis
NC_084178.1 Kalicephalus indicus
PV763276.1 Uncinaria criniformis
NC_026868.1 Strongylus equinus
MW309879.1 Macropostrongyloides dissimilis
OK111103.1 Oesophagostomoides stirtoni
OK111101.1 Oesophagostomoides giltneri
OK111102.1 Oesophagostomoides longispicularis
NC_018602.1 Angiostrongylus vasorum
NC_013067.1 Angiostrongylus costaricensis
AP017675.1 Angiostrongylus costaricensis
NC_030332.1 Angiostrongylus malaysiensis
NC_046586.1 Angiostrongylus mackerrasae
KP721457.1 Angiostrongylus cantonensis
jk2021CZSWF_L5_Q0010W0073_k141_301340
NC_013065.1 Angiostrongylus cantonensis
NC_013065.1 Angiostrongylus cantonensis
MK570632.1 Angiostrongylus cantonensis
MK570631.1 Angiostrongylus cantonensis
Nippostrongylus geno: Cytb
Tree scale 0.009
NC_033886.1 Nippostrongylus_brasiliensis
201709ZJF_k141_283795
jk2021CZSWF_L5_Q0010W0073_k141_335937
201709ZJF_k141_100941
JX877719.1 Tepalcuanema_perezponcedeleoni
AM408289.1 Heligmosomoides_sp._15121Sy
MN953023.1 Heligmosomoides_sp._MSB_Para_RZ1F1
KJ994551.1 Heligmosomoides_neopolygyrus
MN953044.1 Heligmosomum_mixtum
AJ971145.1 Heligmosomoides_costellatum
AJ971146.1 Heligmosomoides_kurilensis_kobayashii
MN953021.1 Heligmosomoides_americanus
AJ971211.1 Heligmosomoides_polygyrus_corsicum
Bootstrap values >90% 70-90%

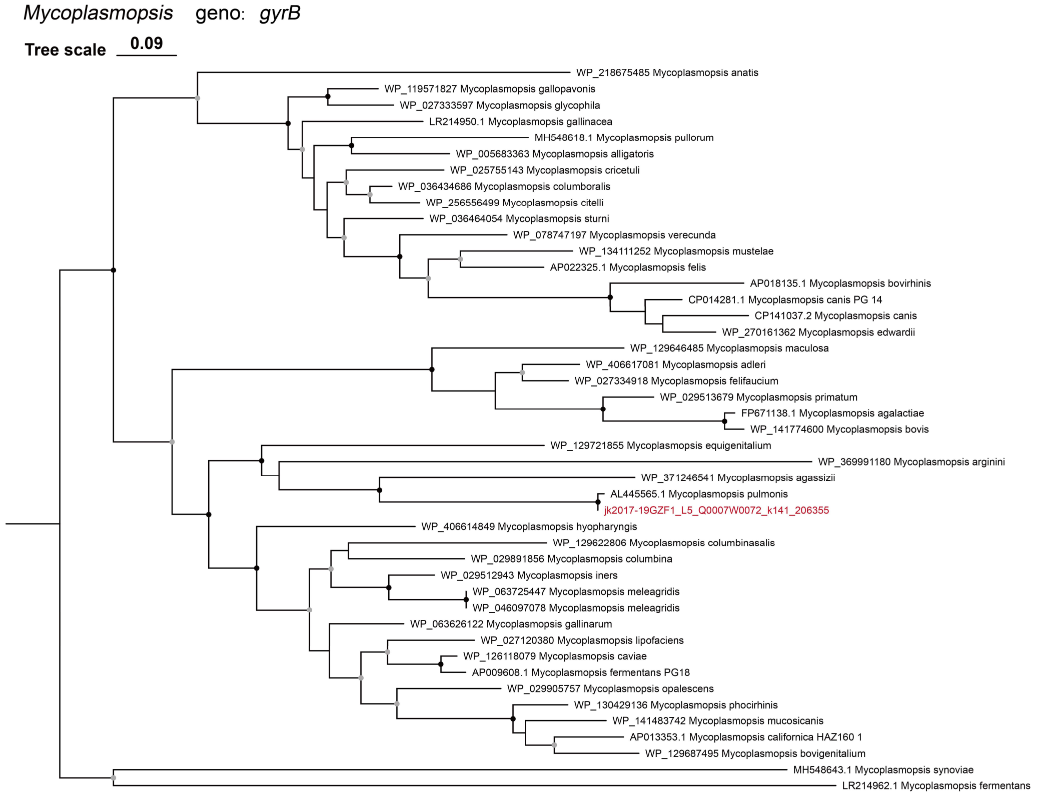

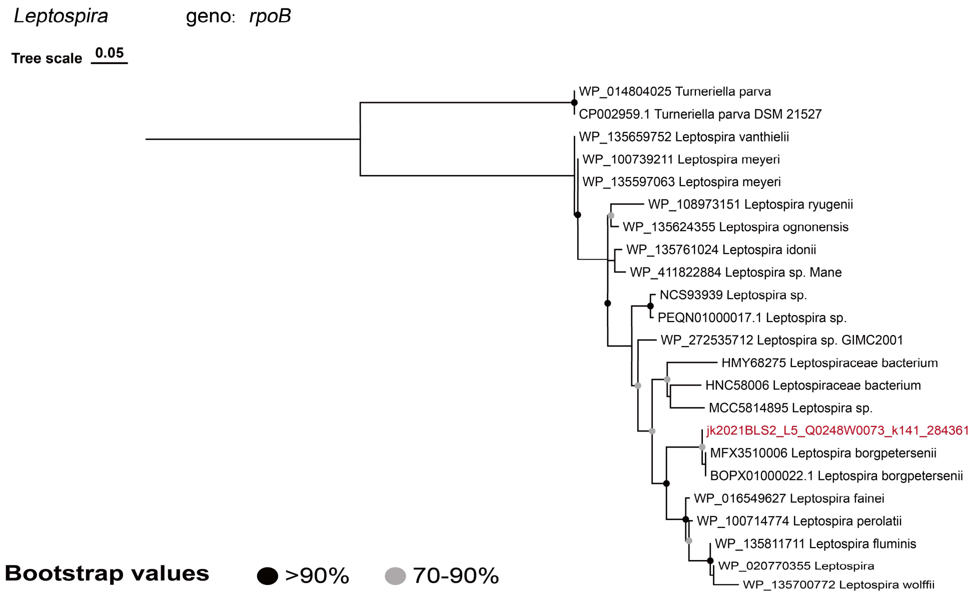

Supplementary Figure S2-S3. Identification of bacterial and eukaryotic pathogens. Each maximum likelihood phylogenetic tree represents the diversity of a pathogen genus and was inferred using the *gyrB* (DNA gyrase subunit B) and *rpoB* (RNA polymerase beta subunit) genes for bacterial pathogens, and *EF1a* (elongation factor 1 alpha), *cox1* (cytochrome c oxidase subunit I), and *Cytb* (cytochrome b) genes for eukaryotic pathogens. Red labels represent pathogens detected in this study and are annotated with their host origins.

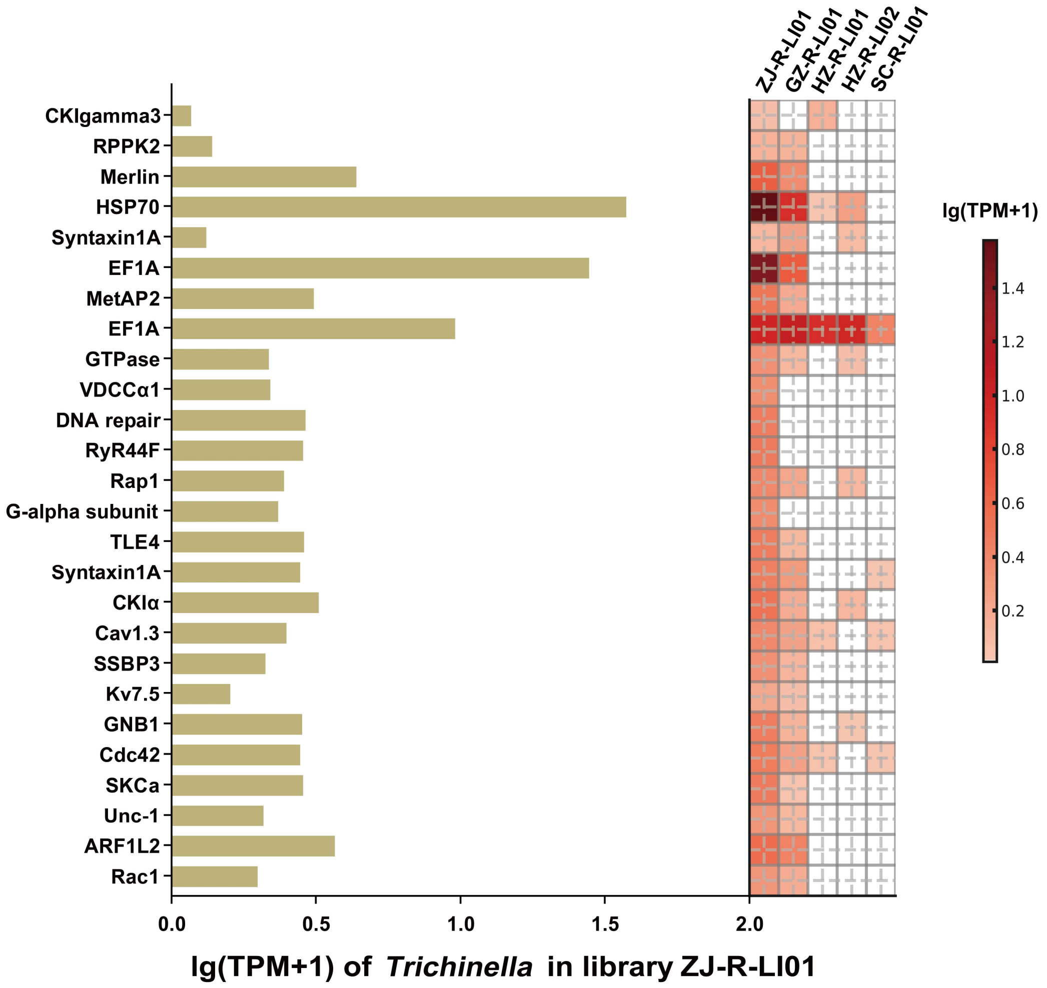

Supplementary Figures S4. 25 expressed genes in libraries with relatively high EF1a abundance (TPM > 5) of Trichinella sp.1

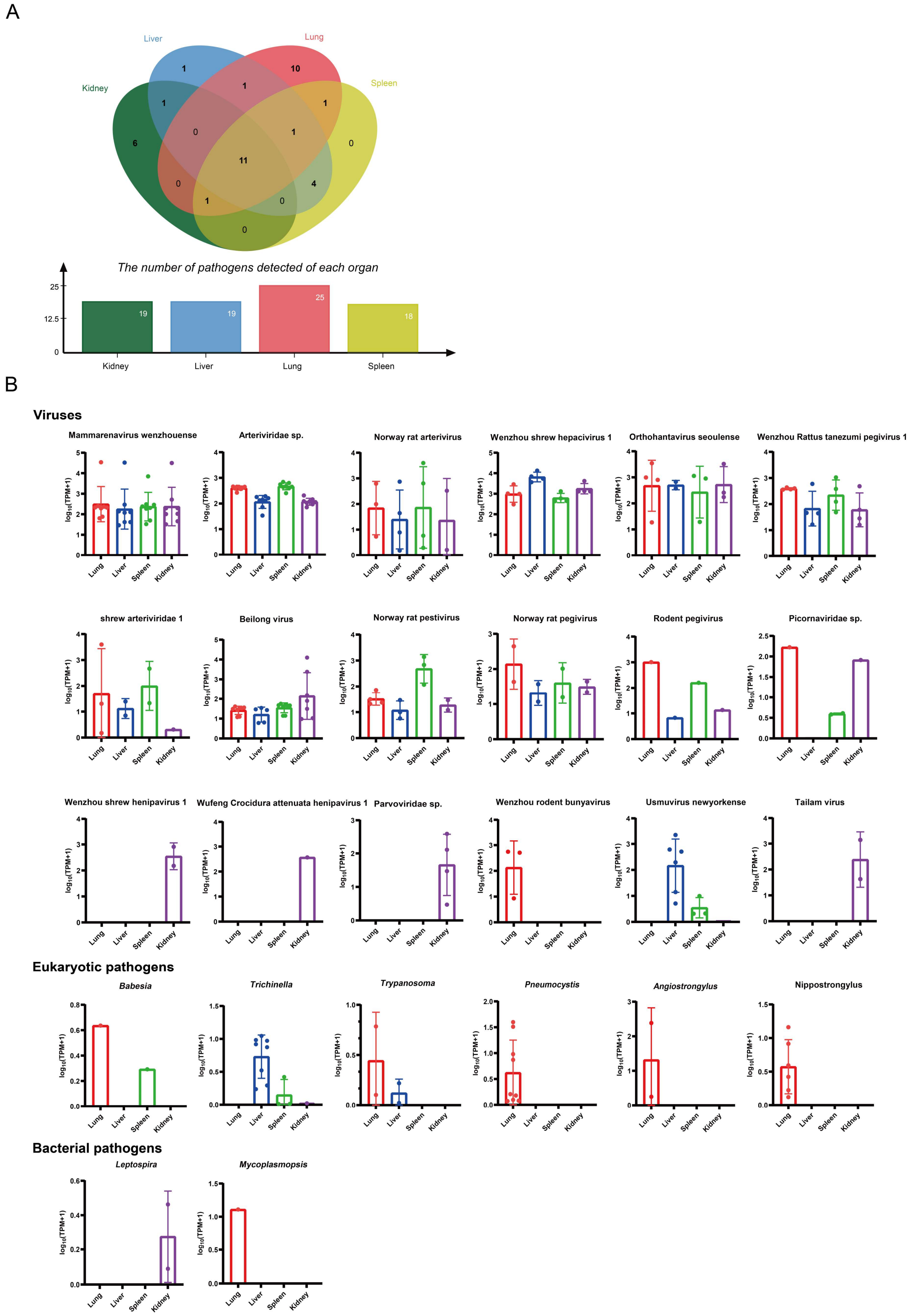

Supplementary Figure S5. Tissue-specific distribution of pathogens (**A**) Venn diagram of pathogen sharing across four organs. (**B**) Tissue distribution of pathogens across host organisms. The charts illustrate the presence and abundance of a subset of the pathogens detected in this study (including viruses, bacteria, and eukaryotes) across different host tissues.
